# The effect of sexual health literacy on sexual function mediated by sexual satisfaction and sexual self-efficacy: Structural equation modeling approach

**DOI:** 10.1371/journal.pone.0318002

**Published:** 2025-01-31

**Authors:** Mojdeh Banaei, Saeed Hosseini, Shiva Alizadeh, Nasibeh Roozbeh, Behnaz Jahanshahloo, Elham Ghasemi, Vida Ghasemi

**Affiliations:** 1 Mother and Child Welfare Research Center, Hormozgan University of Medical Sciences, Bandar Abbas, Iran; 2 Nursing and Midwifery School, Hormozgan University of Medical Sciences, Bandar Abbas, Iran; 3 Department of Midwifery, Zeynab (P.B.U.H) School of Nursing and Midwifery, Guilan University of Medical Sciences, Rasht, Iran; 4 Student Research Committee, Asadabad School of Medical Sciences, Asadabad, Iran; 5 Department of Public Health, Asadabad School of Medical Sciences, Asadabad, Iran; Tarbiat Modares University, ISLAMIC REPUBLIC OF IRAN

## Abstract

Existing literature suggests that sexual health literacy may influence sexual function by enhancing sexual satisfaction and other dimensions of sexual health. This study aimed to investigate the effect of sexual health literacy on sexual function, mediated by sexual satisfaction and sexual self-efficacy, among Iranian women. A cross-sectional study was conducted using structural equation modeling (SEM) with 431 eligible women of childbearing age recruited from health centers in Asadabad city, Hamadan, Iran, from 2023 to 2024 through convenience sampling method. Data were collected using several tools: demographic and obstetric information form, sexual health literacy for adults (SHELA) questionnaire, female sexual function index (FSFI), sexual self-efficacy questionnaire (SSE), and the inventory of sexual satisfaction (ISS). Structural equation modeling was performed using IBM SPSS AMOS version 23. The mean ± SD age of the participants was 31.92 ± 7.24 years. The direct effects of sexual health literacy, sexual self-efficacy, and sexual satisfaction on sexual function were significant (P < 0.001). The indirect effect of sexual health literacy on sexual function, mediated by sexual satisfaction, was also significant (P < 0.001). Overall, the total effect (direct and indirect) of sexual health literacy on sexual function was deemed appropriate, and the proposed conceptual model demonstrated a good fit with the data (χ2/df = 3.35; CFI = 0.966; IFI = 0.967; GFI = 0.951; AGFI = 0.904; RMSEA = 0.070). Based on these findings, sexual health literacy has both direct and indirect effects (mediated by sexual satisfaction) on sexual function. These results may guide professionals in the field of marital relationships to recognize the importance of sexual health literacy and to develop educational or counseling interventions aimed at improving sexual health literacy, ultimately enhancing sexual function.

## Introduction

Sexual health is a fundamental aspect of personal well-being, influencing couples across all ages and life stages [1]. Sexual function, a key domain of sexual health [2] is defined by the absence of difficulties in the phases of sexual desire, arousal, and orgasm, occurring without pain and with subjective satisfaction [3]. Achieving desired and satisfying sexual function positively impacts the quality of sexual life, self-esteem, and interpersonal relationships and has broader implications for overall human well-being [2, 4]. Literature suggests that unmet sexual needs, often due to a lack of skills, information, or awareness, are among the primary contributors to sexual dysfunction [5, 6].

Sexual health literacy, a subset of health literacy, is defined as "a set of knowledge, attitudes, beliefs, motivations, and personal abilities that enable individuals to access, understand, evaluate, and use information related to sexual and reproductive health in everyday life, facilitating discourse, judgment, and decision-making to address sexual concerns and promote sexual health, relationships, and overall well-being" [7, 8]. Numerous studies have indicated that sexual health literacy influences sexual function [6, 8, 9]. By shaping sexual knowledge, attitudes, and beliefs and by empowering individuals to utilize relevant information and services, sexual health literacy plays a crucial role in enhancing sexual satisfaction [6, 10]. Given the established correlation between health literacy and sexual satisfaction [11, 12], it is plausible that sexual health literacy may similarly affect sexual function through the enhancement of sexual satisfaction [10].

Moreover, previous research has underscored the relationship between health literacy and self-efficacy [13, 14]. Improving sexual health literacy can empower individuals to proactively address and resolve sexual health issues, with individuals possessing higher self-efficacy more likely to seek solutions and interventions that enhance their sexual well-being and satisfaction [8, 15].

**Fig 1:**
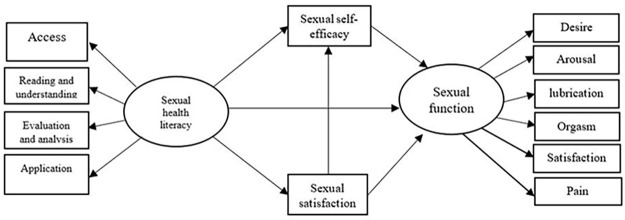
Hypothesized relationships of the research model (Conceptual model).

While earlier studies have explored the associations between sexual health literacy, sexual satisfaction, sexual self-efficacy, and sexual function independently [8–10], to the best of our knowledge, no comprehensive model has been proposed to examine how these variables interact within an integrated framework. Therefore, this study aimed to evaluate the impact of sexual health literacy on sexual function, with sexual satisfaction and sexual self-efficacy as mediators, using a structural equation modeling (SEM) approach in a population of Iranian women. The key innovation of this research lies in its holistic evaluation of the direct and indirect effects of these variables, providing a deeper understanding of the mechanisms through which sexual health literacy influences sexual function (Fig 1). By addressing this gap, our study presents a framework for future research and offers practical insights for interventions designed to improve sexual health outcomes through targeted educational programs.

## Material and method

### Study design and participants

This cross-sectional study was conducted among women of childbearing age who attended health centers in Asadabad city, Hamadan, Iran from 2023 to 2024. The total population of women of childbearing age in Asadabad was 25,614. Based on Cochran’s formula with a 5% margin of error, a ratio of 0.5, and a 95% confidence level, the minimum required sample size was calculated to be 392 participants. To account for a potential 10% attrition rate, the final sample size was set at 431. Ultimately, 431 eligible women were enrolled in the study through a convenience sampling method.

### Inclusion and exclusion criteria

Eligibility criteria for inclusion were: (1) women aged 18 to 45, (2) sexually active with their partner for at least six months, with willing to participate in the study, (3) fluent in Persian, and (4) no history of menopause, pregnancy, or breastfeeding during the study period. Exclusion criteria included having a medical or mental illness, sexual problems, or taking medications known to affect sexual function. Participants with incomplete questionnaire responses were also excluded.

### Procedure

Data was collected between June 12, 2023, and January 15, 2024. Participants were recruited through various channels, including social media, posters, flyers, and verbal. Then, the researcher explained the study’s objectives to women of childbearing age who were referred to health centers in Asadabad. Women who met the inclusion criteria and expressed willingness to participate were informed of the confidentiality measures taken to protect their data, and written informed consent was obtained from them. Subsequently, study data were collected using several standardized questionnaires: the demographic and obstetric information form, the sexual health literacy for adults (SHELA) questionnaire, the female sexual function index (FSFI), the sexual self-efficacy questionnaire (SSE), and the inventory of sexual satisfaction (ISS).

The data collection process took place in a private room during a personal interview with the researcher, ensuring privacy and comfort for the participants. Additionally, the researcher’s contact information was made available to the participants for further inquiries or support related to the study.

### Data collection tools

Data for this study were collected using structured questionnaires, which included the following components:

#### Demographic and obstetric information form

This form comprised 15 questions assessing personal, obstetric, and social characteristics. Items included variables such as age, education level, occupation, spouse’s age, spouse’s education level, income status, duration of marital life, number of live children, parity, and frequency of sexual intercourse per week.

#### Sexual health literacy for adults (SHELA) questionnaire

Developed by Maasoumi et al. in 2018, the SHELA questionnaire evaluates the sexual health literacy of Iranian adults through 40 items. These items are categorized into four domains: access, reading and understanding, evaluation and analysis, and application of information. The total score ranges from 40 to 200 converted to a standard range of 0 to 100 for better interpretation. Scores are classified as follows: 0–50 indicates inadequate sexual health literacy, 50.1–66 represents not so much adequate sexual health literacy, 66.1–84 suggests adequate sexual health literacy, and 84.1–100 denotes excellent sexual health literacy. The validity of this questionnaire was confirmed via face, content, and structure validity, and its reliability was supported by a total Cronbach’s alpha of 0.95, reflecting strong internal consistency. Additionally, the subscales’ intra-class correlation coefficient (ICC) ranged from 0.90 to 0.97, demonstrating the tool’s reliability [16]. In this study, the reliability of the SHELA was recalculated using Cronbach’s alpha test, yielding a value of 0.954, indicating excellent reliability.

#### Sexual self-efficacy questionnaire (SSE)

This questionnaire was developed by Vaziri and Lotfi-Kashani in 2013, based on Schwartzer’s general self-efficacy questionnaire. It consists of 10 items rated on a 4-point Likert scale, ranging from 0 (not true at all) to 3 (completely true). The total possible score ranges from 0 to 30, with higher scores indicating greater self-efficacy. The validity of the questionnaire was established through exploratory factor analysis, and its reliability was assessed using Cronbach’s alpha, yielding a coefficient of 0.851 [17]. In this study, Cronbach’s alpha was recalculated at 0.864, confirming good reliability.

#### Female sexual function index (FSFI)

Developed by Rosen et al. in 2000, the FSFI is widely used for assessing female sexual function. It consists of 19 items across six domains: sexual desire (2 items), sexual arousal (4 items), lubrication (4 items), orgasm (3 items), satisfaction (3 items), and pain (3 items). Most items are rated on a scale from 0 to 5, except for 1, 2, 15, and 16, rated from 1 to 5. A score of zero indicates no sexual activity in the past month. The total possible score ranges from 2 to 36, with higher scores indicating better sexual function [18]. The validity and reliability of the FSFI have been confirmed in several studies [19, 20]. This study calculated Cronbach’s alpha at 0.949, indicating excellent reliability.

#### Inventory of sexual satisfaction (ISS)

Designed by Larson et al. in 1998, the ISS is a 25-item questionnaire that assesses sexual satisfaction using a 5-point Likert scale, ranging from 1 (never) to 5 (always). The total score ranges from 25 to 125, with higher scores indicating greater sexual satisfaction [21]. Previous research, including a study by Bahrami et al., has demonstrated the ISS’s validity and reliability [22]. This study calculated Cronbach’s alpha at 0.773, indicating acceptable reliability.

### Statistical analysis

After data collection, descriptive statistical methods were used to summarize the data. Quantitative variables were presented as means and standard deviations, while qualitative variables were described using frequencies and percentages.

SEM was employed as the primary statistical approach, which is a multivariate regression technique that allows the testing of multiple regression equations simultaneously [23, 24]. This method is particularly useful for examining cause-and-effect relationships between latent and observed variables within a conceptual model [25]. Since normal distribution is a prerequisite for SEM, the normality of the data was assessed through skewness and kurtosis values. The results indicated that the data followed a normal distribution, as the calculated skewness and kurtosis values fell within the acceptable range of -2 to +2 [26].

Confirmatory factor analysis (CFA) was conducted to validate the measurement tools used in the SEM. The reliability of the questionnaires was assessed using several metrics, including the correlation coefficient between observed and latent variables, Cronbach’s alpha (with a minimum acceptable value of 0.70), Composite Reliability (CR > 0.70 indicating acceptable consistency across items), Average Variance Extracted (AVE > 0.50 considered acceptable), and factor loadings (values greater than 0.40 deemed suitable).

SEM was applied to assess both the direct and indirect effects of sexual health literacy on sexual function, with sexual satisfaction and sexual self-efficacy as moderating variables. Various fit indices were used to evaluate the goodness-of-fit of the SEM model, including the normed chi-square (χ^2^/df), comparative fit index (CFI), goodness-of-fit index (GFI), incremental fit index (IFI), normal fit index (NFI), and root-mean-square error of approximation (RMSEA) [27].

All SEM analyses were performed using IBM SPSS AMOS version 23. A P-value of less than 0.05 was considered statistically significant for all analyses.

### Ethical consideration

This study was approved by the Ethics Committee of Asadabad School of Medical Sciences (Ethical code: IR.ASAUMS.REC.1402.004). Confidentiality of the collected data was ensured, and written informed consent was obtained from all research participants. Participants were also free to decline participation or withdraw at any stage of the research process.

## Result

### Demographic and sexual characteristics

A total of 431 women of childbearing age participated in this study. The mean age of the participants was 31.92 ± 7.24 years. Nearly half of the participants (47.1%) held a university degree, and the majority (63.1%) identified as housewives. The average duration of marriage was 9.41 ± 7.08 years, ranging between 1 and 32 years. The majority of participants (83.3%) reported having sexual intercourse 1–3 times per week. The most common source of sexual information, cited by 58% of the participants, was mass media, including the internet, social media, radio, and television. Further demographic and sexual characteristics are presented in Table 1.

**Table 1:** Socio-demographic, obstetric, and sexual characteristics (n = 431).

Variables		N (%) / Mean ± SD
Spouse’s age		36.67±7.44
Educational level	Primary	23 (5.3)
	Secondary	59 (13.7)
	High school and diploma	146 (33.9)
	University	203 (47.1)
Spouse’s education level	Primary	17 (3.9)
	Secondary	79 (18.3)
	High school and diploma	147 (34.1)
	University	188 (43.6)
Economic status	Satisfied	51 (19.6)
	Intermediate	280 (65)
	Dissatisfied	75 (17.4)
Occupation	Housewife	272 (63.1)
	Employee	159 (36.9)
Marriage duration (years)		9.41±7.08
Contraception method	Hormonal	83 (19.3)
	Non-hormonal	139 (32.3)
	Others	209 (48.5)
Number of parity		1.38 ±1.20
Number of children		1.18±0.94
Current use of antidepressants	Yes	26 (6)
	No	405 (94)
Private location for the sexual relationship	Yes	391 (90.7)
	No	40 (9.3)
Sexual intercourse per week	1–3 times	359 (83.3)
	4–6 times	66 (15.3)
	≥ 7 times	6 (1.4)
Anal sex	Yes	129 (29.9)
	No	302 (70.1)
Source of sexual information	Friends	99 (23)
	Family	68 (15.8)
	Books	97 (22.5)
	Health providers	198 (45)
	Mass-media	250 (58)
	None	50 (11.6)

SD = standard deviation

The mean ± SD total score for sexual health literacy was 71.24 ± 15.06, with scores ranging from 0 to 100. The majority of participants (n = 192, 44.5%) had adequate sexual health literacy, while 115 (26.7%) had not so much adequate sexual health literacy, 90 (20.9%) demonstrated excellent sexual health literacy, and 34 (7.9%) had inadequate sexual health literacy. The mean ± SD total score for sexual function was 26.20 ± 6.29 out of a maximum score of 36, with 52.2% of the women reporting unfavorable sexual function (FSFI<28). Table 2 presents the mean ± SD scores for sexual self-efficacy and sexual satisfaction, the skewness, and kurtosis indices for the latent and observed variables in the SEM model. As shown in Table 2, the skewness and kurtosis indices fall within the acceptable range (-2 to 2), indicating that the data are normally distributed.

**Table 2:** Statistical summary of observed and latent variables (n = 431).

Factor	Mean	SD	Skewness	Kurtosis
**Sexual Function**	26.20	6.29	-1.152	1.691
Desire	4.06	1.06	-0.33	0.12
Arousal	4.06	1.20	-0.66	.70
Lubrication	4.46	1.27	-0.97	.85
Orgasm	4.54	1.38	-1.21	1.28
Satisfaction	4.68	1.47	-1.04	.02
*Pain*	4.41	1.40	-0.99	.94
**Sexual Health Literacy**	71.24	15.06	-.114	-.104
Access	71.15	19.40	-0.56	.57
Reading and understanding	73.30	16.00	-0.23	-.07
Evaluation and Analysis	68.67	19.21	-0.22	.07
Application of information	71.88	16.37	-0.25	-.33
**Sexual Satisfaction**	101.04	14.29	-1.06	.75
**Sexual Self-efficacy**	17.69	5.69	-0.19	.06

SD = standard deviation, Min = minimum, max = maximum.

### Assessment of measurement model

As shown in Table 3, there were significant positive correlations between sexual health literacy, sexual satisfaction, and sexual self-efficacy with sexual function. Cronbach’s alpha values for the constructs ranged from 0.773 to 0.954, indicating strong internal consistency. The CR scores ranged from 0.761 to 0.880, exceeding the recommended threshold of 0.70 for all constructs. Additionally, the AVE for each construct was greater than 0.50, demonstrating acceptable convergent and discriminant validity. This indicates that the model’s constructs were consistent and distinct, providing evidence of good measurement validity.

**Table 3:** Correlation coefficients matrix, Cronbach’s Alpha, CR, and AVE scores of the scales.

Factor	Sexual Function	Sexual Health Literacy	Sexual Satisfaction	Sexual Self-efficacy	Cronbach’s Alpha	CR	AVE	R square (%)
Sexual Function	1	-	-	-	0.949	0.831	0.57	65
Sexual Health Literacy	.420**	**1**	-	-	0.954	0.874	0.64	-
Sexual Satisfaction	.625**	.445**	1	-	**0.773**	0.761	0.611	23
Sexual Self-efficacy	.454**	.365**	.423**	1	0.864	0.880	0.543	22

*P<0.05;

**P<0.01;

***P<0.001;

CR = Composite Reliability; AVE = Average Variance Extracted.

**Fig 2:**
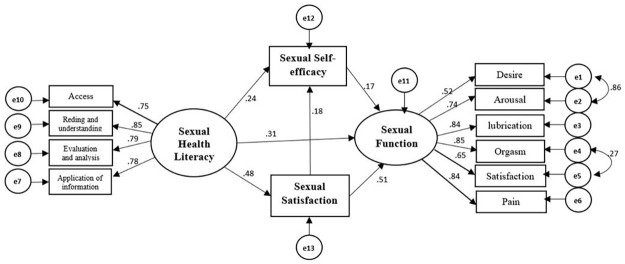
The final model of sexual health literacy and its relationship with sexual function, satisfaction, and self-efficacy.

As shown in Fig 2, the factor loadings (path coefficients) for the four subscales of the sexual health literacy questionnaire ranged from 0.75 to 0.85, and the factor loadings for the six domains of the FSFI varied between 0.52 and 0.85. These values are all greater than 0.4, indicating that the factors are appropriate for assessing the validity of the questionnaires.

R-squared (R^2^) values were used to evaluate the SEM model’s explanatory power for independent variables. In our model, the R^2^ values for sexual self-efficacy (22%) and sexual satisfaction (23%) were moderate (above 19%), while the R^2^ value for sexual function (65%) indicated a strong explanatory power (above 60%) (Table 3).

### SEM model

The initial model was constructed based on the theoretical assumptions outlined in the introduction (Fig 1). Fig 2 and Table 4 present the results of both the direct and indirect effects of sexual health literacy on sexual function, with sexual self-efficacy and sexual satisfaction as moderating variables. The direct effects of sexual health literacy, sexual self-efficacy and sexual satisfaction on sexual function were all statistically significant (P < 0.001).

**Table 4:** Standardized direct and indirect effects of sexual health literacy on sexual function.

Effect	Pathway	Factor loading	P value
Direct effects	sexual health literacy → sexual function	0.31	<0.001
	sexual self-efficacy → sexual function	0.17	<0.001
	sexual satisfaction → sexual function	0.51	<0.001
Indirect effects	sexual health literacy → sexual self-efficacy → sexual function	0.24×0.17 = **0.041**	0.271
	sexual health literacy → sexual satisfaction → sexual function	0.48×0.51 = **0.245**	<0.001
	sexual health literacy → sexual satisfaction → sexual self-efficacy → sexual function	0.48×0.18×0.17 = **0.015**	0.509

The indirect effects of sexual health literacy on sexual function are summarized across three pathways, as shown in Table 4.

#### First pathway (Sexual health literacy → Sexual self-efficacy → Sexual function)

When sexual self-efficacy is the moderating variable, the indirect effect of sexual health literacy on sexual function is calculated by multiplying the direct effect of sexual health literacy on sexual self-efficacy (0.24) by the effect of sexual self-efficacy on sexual function (0.17): 0.24 × 0.17 = 0.041. The results indicated that this effect was weak and not statistically significant (P = 0.271).

#### Second pathway (Sexual health literacy → Sexual satisfaction → Sexual function)

When sexual satisfaction is the only moderating variable, the indirect effect of sexual health literacy on sexual function is calculated as 0.48 × 0.51 = 0.245. This pathway revealed that the indirect effect of sexual health literacy on sexual function, mediated by sexual satisfaction, was significant (P < 0.001).

#### Third pathway (Sexual health literacy → Sexual satisfaction → Sexual self-efficacy → Sexual function)

As shown in Table 4, the indirect effect for this pathway is calculated as 0.48 × 0.18 × 0.17 = 0.015. Thus, the indirect effect of sexual health literacy on sexual function in this pathway was weak and not statistically significant (P = 0.509).

The overall effect of sexual health literacy (the sum of direct and indirect effects) on sexual function was calculated as follows: (0.041 + 0.245 + 0.015) + 0.31 = 0.611. Given that this result is greater than 0.4, it was considered appropriate [27].

Table 5 presents the results for all goodness-of-fit indices for our model. These findings indicate that the proposed conceptual model fits the data well.

**Table 5:** The goodness of fit index.

Fit Index	*χ*^2^/*df*	TLI	CFI	IFI	NFI	GFI	AGFI	RMSEA	SRMR
Acceptable Threshold Levels	<5	>0.95	>0.90	>0.90	>0.95	>0.95	>0.80	<0.08	<0.08
Estimated value	3.35	.954	.966	.967	.953	.951	.904	.070	.0.035
Result	Reasonable	Reasonable	Reasonable	Reasonable	Reasonable	Reasonable	Reasonable	Reasonable	Reasonable

TLI, Tucker Lewis index; CFI, comparative fit index; GFI, goodness-of-fit index; IFI, incremental fit index; NFI, normal fit index; AGFI, adjusted goodness-of-fit index; RMSEA, root mean square error of approximation; SRMR, standardized root means square Residual

## Discussion

This study aimed to evaluate the effect of women’s sexual health literacy on sexual function, focusing on the mediating roles of sexual self-efficacy and sexual satisfaction. SEM was employed, and the results demonstrated a good model fit. In summary, the findings indicate that sexual health literacy, sexual self-efficacy, and sexual satisfaction each have a direct and positive effect on sexual function. The indirect effect of sexual health literacy on sexual function was significant only when sexual satisfaction was the moderating variable but not when sexual self-efficacy was considered the moderator. Nevertheless, the overall effect of sexual health literacy on sexual function, both direct and indirect, was appropriate.

Sexual health literacy encompasses a range of skills, abilities, and knowledge that can positively influence sexual health outcomes [28]. This study’s results revealed that most participants (44.5%) had adequate sexual health literacy. This is consistent with findings from a study conducted in Qazvin by Panahi et al., where 50.1% of participants had adequate sexual health literacy [29]. The relationship between education level and sexual health literacy may help explain these results [29], as nearly half of the participants in our study held university degrees.

Additionally, our research revealed that among the four dimensions of sexual health literacy, the highest mean score was observed in the "reading and understanding" dimension, while the lowest mean score was in the "evaluation and analysis" dimension. Dabiri et al.’s study similarly found that the highest health literacy scores were associated with reading and understanding information, while the lowest scores pertained to access to information [30]. The differences in findings may be attributed to the influence of the research environment on individuals’ access to sexual information. Access to accurate and specific sexual health information can be challenging and is often influenced by factors such as formal education, provider awareness, parental education, and societal taboos or shame.

Sexual function is a critical aspect of human life, influenced by various factors such as personal characteristics, educational attainment, interpersonal relationships, family dynamics, socio-cultural conditions, physical and psychological health, and hormonal status [31, 32]. One key factor that has been shown to improve sexual function is sexual health literacy [10]. Our study’s results confirm a significant direct effect of sexual health literacy on sexual function. In line with these findings, Dehghankar et al. reported a significant correlation between sexual health literacy and sexual function, demonstrating that women with excellent, sufficient, or not enough sexual health literacy had 4.222, 2.219, and 1.313 times higher odds of optimal sexual function, respectively, compared to those with inadequate health literacy [9]. Improving sexual health literacy can significantly reduce sexual problems. As individuals gain greater understanding and knowledge, they are better equipped to apply this information to enhance their physical and mental well-being during sexual activity, ultimately leading to improved sexual function [6, 10].

In this study, sexual self-efficacy and sexual satisfaction served as mediating variables within the SEM framework. The findings revealed a significant direct effect of sexual self-efficacy on sexual function. According to Bandura (1977), developing strong self-efficacy beliefs is just as crucial as acquiring literacy skills. Individuals who are competent and confident in their literacy abilities are more likely to apply those skills and solve problems [33, 34]. Similarly, sexual self-efficacy can foster healthier sexual relationships, promote healthy sexual behaviors, and enhance both sexual and general health, making it essential for proper sexual function [35, 36].

A study by Atrian et al. examined the relationship between sexual self-efficacy and sexual function in married women, finding that sexual self-efficacy was closely linked to sexual function and several of its subscales, including libido, orgasm, lubrication, and sexual arousal [37]. Additionally, multiple studies have shown that individuals with higher sexual self-efficacy are more likely to seek treatment [38], address sexual problems, and engage in sexual self-care [39]. This association was also confirmed by Ghasemi et al. in a study on patients with multiple sclerosis [40].

Despite the significant direct effect of sexual self-efficacy on sexual function, the indirect effect of sexual health literacy on sexual function, when sexual self-efficacy was the mediating variable, was not significant. However, this indirect effect was significant when sexual satisfaction was the mediating variable. Sexual satisfaction refers to the overall positive feelings an individual has about their sexual experiences [41], which are influenced by their psychological state, attitudes toward sexual relations, and the level of pleasure derived from those experiences [42].

The results of the present study showed that the mean score for sexual satisfaction was favorable, and the direct effect of sexual satisfaction on sexual function was significant. Similarly, Ziaei et al. found a significant positive correlation between sexual satisfaction and sexual function [41]. In other words, higher sexual satisfaction may result in fewer complaints from women experiencing sexual disorders [43]. According to Rowland (2015), positive aspects of sexual self-concept, including sexual self-esteem, sexual satisfaction, and sexual self-efficacy, are closely related to sexual function [44].

Furthermore, the study results demonstrated that the effect of sexual health literacy on sexual function, with sexual satisfaction as a mediating variable, was significant. Sahebalzamani et al. found that health literacy was more strongly associated with higher levels of sexual function and satisfaction in both men and women [10]. It can be concluded that increasing sexual health literacy by enhancing an individual’s skills in addressing sexual issues can positively influence the mood and attitudes of couples towards sexual and marital matters, thereby improving sexual satisfaction and, consequently, sexual function [9].

### Strengths and limitations

One of this study’s strengths is its use of a path analysis model. Path analysis allows for the simultaneous examination of multiple variables, a capability not available in simpler statistical models, making this a significant advantage. Another strength of the present study was the use of standardized questionnaires.

However, one limitation of this study is that sexual health literacy is a relatively new subject, and to our knowledge, no research has applied path analysis to this topic. As a result, it is challenging to compare our findings with those of similar studies due to the lack of comparable research. The cross-sectional nature of this study also limits the ability to establish a causal relationship between variables. Additionally, information bias may arise due to the sensitive nature of sexual health topics. To mitigate this bias, participants were assured that their data would remain confidential.

The present study was conducted only on women of reproductive age in urban health centers. Hence, a population-based study with a larger sample size, in a comparative design between urban and rural populations and with the participation of both partners, is suggested.

## Conclusion

Based on the findings of this study, sexual health literacy had both direct and indirect effects (mediated by sexual satisfaction) on sexual function. The overall effect of sexual health literacy on sexual function was substantial. Given the importance of sexual health literacy, its role is critical in enhancing individual sexual well-being and, ultimately, improving family and social health. Therefore, these findings can assist professionals working in marital and relationship counseling to recognize the importance of sexual health literacy and to design and implement educational or counseling interventions aimed at improving sexual health literacy, which in turn could enhance sexual function.
